# Usefulness of the Heavy Menstrual Bleeding Diagnostic Tools SAMANTA Questionnaire and Heavy Menstrual Bleeding–Visual Analog Scale Tool for Iron Deficiency Screening: An Exploratory Analysis from the COLIBRI Study

**DOI:** 10.1177/26884844251382731

**Published:** 2025-09-26

**Authors:** Josep Perelló-Capó, Josep Estadella-Tarriel, Ignasi Gich-Saladich, Elisa Llurba-Olivé, Joaquim Calaf-Alsina

**Affiliations:** ^1^ Department of Obstetrics and Gynecology, Hospital de la Santa Creu i Sant Pau, Barcelona, Spain.; ^2^ Universitat Autònoma de Barcelona, Barcelona, Spain.; ^3^ Clinical Epidemiology and Public Health Service, IIB Sant Pau, Universitat Autònoma de Barcelona, Barcelona, Spain.; ^4^ Primary Care Interventions to Prevent Maternal and Child Chronic Diseases of Perinatal and Developmental Origin Network (RICORS), Instituto de Salud Carlos III, Madrid, Spain.

**Keywords:** heavy menstrual bleeding, iron deficiency, quality of life, screening, ferritin, performance

## Abstract

**Background:**

The SAMANTA questionnaire and the Heavy Menstrual Bleeding–Visual Analog Scale (HMB-VAS) tool have been validated for diagnosing heavy menstrual bleeding (HMB). We assessed their value for screening iron deficiency (ID).

**Material and Methods:**

Post hoc analysis of the prospective, randomized, phase 4 Cooper and Levonorgestrel Intrauterine Device (IUD) Barcelona Research Iniciative (COLIBRI) study, which assessed the bleeding profile of two intrauterine devices. We used information collected during the last follow-up visit (month 36): sociodemographics, hemoglobin/ferritin levels, SAMANTA questionnaire, HMB-VAS tool, and EuroQoL five-dimension five-level scores. The primary outcome was the accuracy of these diagnostic tools in identifying ID. We also assessed their relationship with EQ-5D-5L.

**Results:**

We analyzed information from 57 women, 18 (31.6%) and 14 (24.6%) with HMB according to the SAMANTA questionnaire and the HMB-VAS tool, respectively. Ferritin levels showed better inverse correlation than hemoglobin with these HMB diagnostic tools’ scores: *r* = −0.539 and *r* = −0.557, respectively, both *p* < 0.001. In women with HMB according to these tools, the ferritin threshold showing the best sensitivity and specificity to identify ID was <10 ng/mL with the SAMANTA questionnaire (71.4% [Confidence Interval (CI) 95% 35.2–93.5] and 78% [CI 95% 65.2–87.7], respectively) and <15 ng/mL for the HMB-VAS tool (58.3% [CI 95% 31.2–87.7] and 82.2% [CI 95% 69.2–91.2], respectively). Only the EQ-VAS scores correlated inversely with the scores of both HMB diagnostic tools (*r* = −0.308, *p* = 0.02, and *r* = −0.294, *p* = 0.026, respectively).

**Conclusions:**

This exploratory analysis shows the SAMANTA questionnaire’s good potential for ID screening. The scores of both HMB diagnostic tools correlated with the EQ-VAS, demonstrating their value in capturing the HMB impact on quality of life.

## Introduction

Heavy menstrual bleeding (HMB) is defined as excessive menstrual blood loss that adversely impacts women’s physical, emotional, social, and material quality of life (QoL), which can occur alone or in combination with other symptoms.^1^ This condition is probably underdiagnosed due to several factors, among which is the complexity in using currently available diagnostic methods in clinical practice.^2,3^ Easy-to-use tools addressing HMB’s quantitative and qualitative aspects, such as the 6-item SAMANTA questionnaire^4,5^ and the HMB-Visual Analog Scale (HMB-VAS)^5,6^ are useful to improve HMB identification.

The SAMANTA questionnaire allows the screening of women with excessive menstrual loss interfering with QoL with a sensitivity of 86.7% and a specificity of 89.5%.^4,5^ It has also been validated for monitoring HMB hormonal treatment.^7^ Similarly, the HMB-VAS, which measures bleeding intensity and impact, has also shown good performance for HMB screening, with a sensitivity of 89.6% and a specificity of 85%.^5,6^ It is worth noting that despite including QoL assessment, the correlation of these tools with those used for assessing QoL had not been investigated.

Part of the impact of HMB on daily activities detected by these HMB diagnostic tools may be explained by the physical and cognitive impairment^8–11^ caused by iron deficiency (ID), independently of iron deficiency anemia (IDA) due to heavy menstrual blood loss.^12,13^ Despite the importance of an early diagnosis of ID/IDA in patients with HMB, screening and treatment of these conditions are hampered by the lack of consensus.^9^ Anemia has classically been diagnosed according to serum hemoglobin (Hb) levels (Hb < 120 g/L in nonpregnant females).^14^ However, the sensitivity and specificity of Hb in detecting ID are low.^15,16^ Moreover, common symptoms of anemia, such as fatigue, may appear without anemic Hb levels.^17^ Conversely, serum ferritin level, an indicator of iron stores, is a more sensitive and specific biomarker for assessing ID.^18^ However, ferritin assessment is not standardized, and the limitations associated with its measurement preclude its wide use.^9^

Given that the SAMANTA questionnaire and the HMB-VAS tool assess the bleeding intensity and the impact on QoL, we evaluated whether these HMB diagnostic tools could help screen for ID. For this purpose, we used data from the last follow-up visit of the Cooper and Levonorgestrel Intrauterine Device (IUD) Barcelona Research Iniciative (COLIBRI) study—which assessed the bleeding profile and safety of a levonorgestrel 13.5 mg intrauterine device (LNG13.5-IUD) versus a Nova T copper 380 mm^2^ IUD (Cu380-IUD) during 3 years—given that investigators were asked to use the SAMANTA questionnaire and the HMB-VAS tool to assess HMB and to analyze Hb and ferritin levels.^19^ As information on QoL was also collected using the EuroQoL five-dimension five levels (EQ-5D-5L), we also assessed the correlation of the score of these HMB tools with QoL as assessed by this generic instrument.

## Materials and Methods

### Data source

The present study is a post hoc analysis of the prospective, randomized, phase 4 COLIBRI study (Cooper and Levonorgestel IUD Barcelona Research Initiative; EudraCT No. 2015-004956-23; NCT02957292). This study was conducted between March 2016 and September 2020 at the Santa Creu and Sant Pau Hospital (Barcelona, Spain). The design of the COLIBRI study has been described elsewhere.^19^ Briefly, women aged 18–45 years attending the Department of Obstetrics and Gynecology looking for contraceptive methods were allowed to participate if they did not wish to become pregnant in the next 3 years, had regular periods, did not experience severe dysmenorrhea, and requested an LNG13.5/Cu380-IUD after adequate counseling. The main exclusion criteria included having absolute contraindications to using an LNG/Cu-IUD, menstrual bleeding irregularities, or anemia.

### Data collection

We used data collected during the final visit of the COLIBRI study, which took place 36 months after the IUD insertion. It included socio-demographic information, the HMB diagnostic tools SAMANTA questionnaire and HMB-VAS scores, the EuroQoL five-dimension five-level (EQ-5D-5L) ratings, and serum Hb and ferritin levels from a blood sample drawn during the same week.

### Outcomes

The primary outcome was the accuracy (sensitivity and specificity) of the SAMANTA questionnaire and the HMB-VAS tool in identifying ID. For this purpose, the relationship between the scores of these HMB diagnostic tools and serum ferritin and Hb levels was first analyzed. The HMB diagnostic tool and the blood parameter showing a better relationship in this analysis were selected for ID identification performance assessment. The secondary outcome was the relationship between the scores of these HMB diagnostic tools and the EQ-5D-5L scores.

### Measurements

HMB was assessed with the HMB tools, SAMANTA questionnaire, and HMB-VAS. The SAMANTA questionnaire consists of six questions: (1) Generally, do you bleed for more than 7 days a month? (2) Do you have 3 or more days of increased heavy bleeding during your period? (3) In general, do you find your periods particularly inconvenient due to their heaviness? (4) On any of the heavier bleeding days, do you bleed and stain your nightwear, or would you stain it if you did not use double protection or change during the night? (5) During heavier bleeding days, do you worry about staining the seat of your chair, sofa, etc.? (6) In general, on heavier bleeding days, do you avoid (if possible) certain activities, travel, or leisure plans because you need to change your tampon or pad frequently? Affirmative answers to items 1 and 3 are scored 3, while the remaining items are scored 1. Negative answers score 0. The total score ranges from 0 to 10, with a score of ≥3 points indicative of HMB.^4,5^

The total score of the HMB-VAS screening tool is calculated using the scores of the intensity of menstrual bleeding (VASInt) and its impact on activities of daily living (VASImp) by the following function: 11xVASInt score + 2xVASImp score. VASInt and VASImp scores are obtained by making a handwritten mark on a 100-mm horizontal line on the same chart. For VASInt, the line represents a continuum from “No bleeding at all” to “The heaviest possible menstrual bleeding I have had,” scoring 0 and 100, respectively. For VASImp, the line represents a continuum from “It does not interfere with my daily life/activities at all’’ to ‘‘Completely interferes in my daily life/activities,” scored 0 and 100, respectively. A total score of ≥700 is indicative of HMB.^5,6^

We used the validated Spanish version of the EQ-5D-5L for the assessment of QoL.^20^ This tool comprises a descriptive system and a EuroQoL Visual Analog Scale (EQ-VAS). The descriptive questionnaire evaluates five health-related dimensions (mobility, self-care, usual activities, pain/discomfort, and anxiety/depression), each with five response levels (no problem, slight problems, moderate problems, severe problems, unable to/extreme problems). Responses are coded as single-digit numbers expressing each dimension’s severity level. An EQ-5D-5L summary index is obtained by using a scoring algorithm that attaches values (weights) to each of the levels in each dimension according to a national value set. The EQ-VAS is a patient-reported quantitative measure that consists of a 200-mm vertical line where endpoints represent a range of assessments from “The best health you can imagine” to “The worst health you can imagine,” scored 100 and 0, respectively.^21,22^

### Statistical analysis

Quantitative variables are presented as the median and the 25th and 75th percentiles (interquartile range [IQR]). We used Spearman’s correlation coefficient to analyze the correlation between the SAMANTA questionnaire and HMB-VAS scores and ferritin and Hb serum levels on the one hand and with EQ-5D-5L scores on the other. To assess the diagnostic ability of the SAMANTA questionnaire and the HMB-VAS to identify cases of ID among those with an HMB diagnosis according to these tools, we constructed a receiver operating characteristic (ROC) curve by plotting the sensitivity (or true positive rate) against the specificity (1—false positive rate). Three ferritin thresholds were used: <10, <15, and <30 ng/mL. We calculated the area under the ROC curve (AUC) to quantify the overall discriminative ability of the model. The Youden’s index (Sensitivity + Specificity − 1) was calculated for each threshold to identify the optimal decision threshold (best balance between sensitivity and specificity). The analyses were performed with the IBM-SPSS version 26 statistical package and considered a two-sided *p* value <0.05 as significant.

### Ethics statements

The study was approved by the Santa Creu and Sant Pau Hospital Ethics Committee and conducted following the Declaration of Helsinki and the International Conference on Harmonization—Good Clinical Practice guidelines. Participants provided written informed consent before participation. The report of this subanalysis follows the Strengthening the Reporting of Observational Studies in Epidemiology (STROBE) guidelines, available at https://www.equator-network.org.

## Results

### Subjects

Fifty-seven women were included in this analysis (all the women participating in the study at this stage, except one who refused to participate). Of these, 28 (49.1%) were Cu380-IUD users, and 29 (50.9%) were LNG13.5-IUD users. Their mean age was 32.8 ± 7.3 years (Table 1). HMB was diagnosed in 18 (31.6%) women, according to the SAMANTA questionnaire, and in 14 (24.6%) women, according to the HMB-VAS tool. In women with ferritin <30 ng/mL (*n* = 22), HMB was identified in 12 (54.5%) with the SAMANTA questionnaire and 11 (50%) by the HMB-VAS tool. In women with ferritin <15 ng/mL (*n* = 12), HBM was identified in 8 (66.7%) and 7 (58.3%), respectively. In women with ferritin <10 ng/mL, it was identified in 5 (71.4%) and 4 (57.1%), respectively.

**Table 1.** Baseline Characteristics of Women Included in the Analysis

Age, years, mean (SD)	32.8 (7.3)
BMI, kg/m^2^, mean (SD)	25.4 (4.9)
Education level, *n* (%)	
University	19 (33.3)
Secondary school	34 (59.6)
Primary school	3 (5.3)
None	1 (1.8)
Socioeconomic level^a^, *n* (%)	
High	0 (0)
Medium-high	6 (10.5)
Medium	42 (73.7)
Medium-low	7 (12.3)
Low	2 (3.5)
Nulliparous, yes, *n* (%)	19 (33.3)

^a^ Self-reported.

BMI, body mass index; SD, standard deviation.

### Ferritin and Hb serum levels in women with and without HMB

**FIG. 1.**
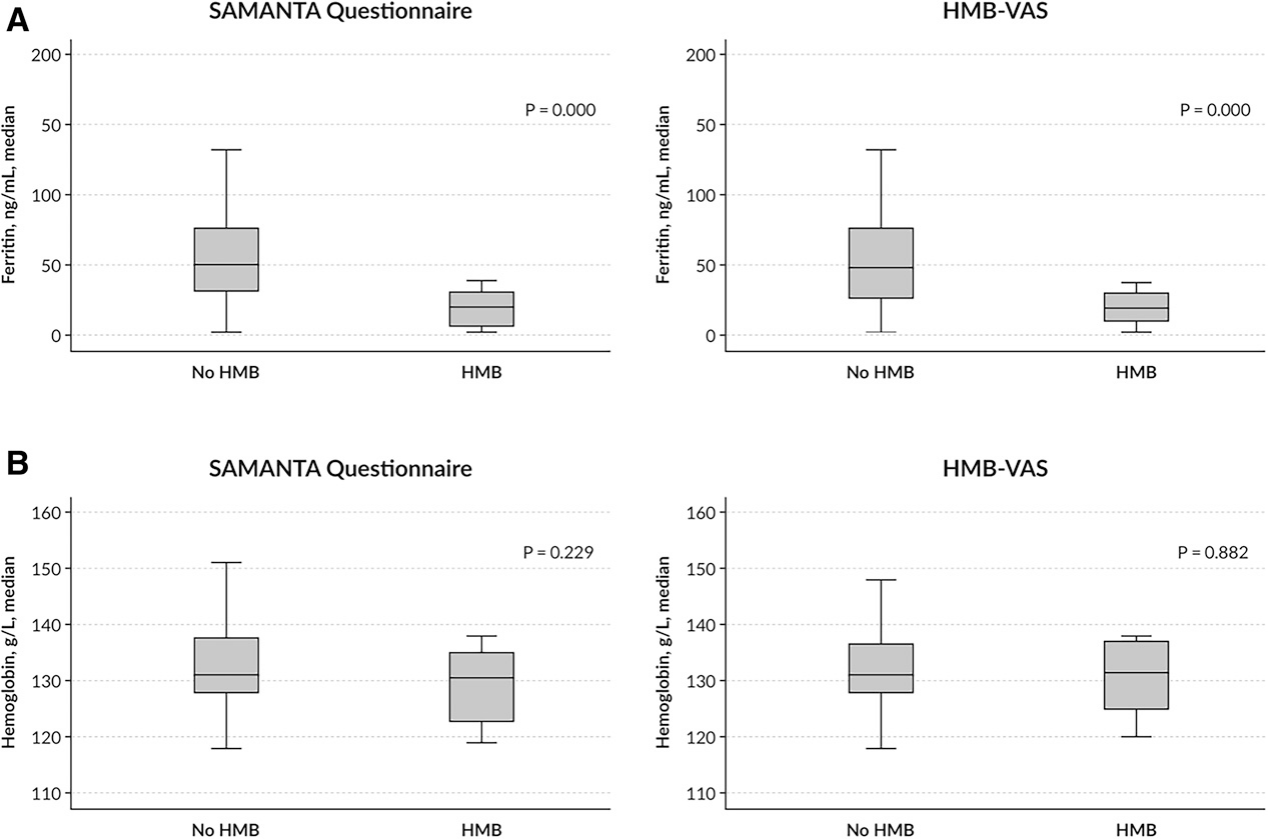
Median serum ferritin **(A)** and hemoglobin **(B)** levels in women with or without HMB according to the diagnostic tools SAMANTA questionnaire and HMB-VAS. HMB, heavy menstrual bleeding; HMB-VAS, Heavy Menstrual Bleeding–Visual Analog Scale; SAMANTA-Q, SAMANTA questionnaire.

The median serum ferritin levels were significantly lower in women with HMB versus those without HMB according to the SAMANTA questionnaire (23.1 ± 19.6 vs. 58.5 ± 37.7 ng/mL, *p* < 0.001) and according to the HMB-VAS tool (19.6 ± 11.5 vs. 56.4 ± 37.8 ng/mL, *p* < 0.001), both respectively (Fig. 1A). No difference was observed in the median serum Hb level in women with and without HMB according to the SAMANTA questionnaire (130.5 [122.7–135.5] vs. 131.0 [128.0–138.0] g/L, *p* = 0.229) and the HMB-VAS tool (131.5 [124.5–137.0] vs. 131.0 [128.0–137.0] g/L, *p* = 0.882), both, respectively (Fig. 1B).

**FIG. 2.**
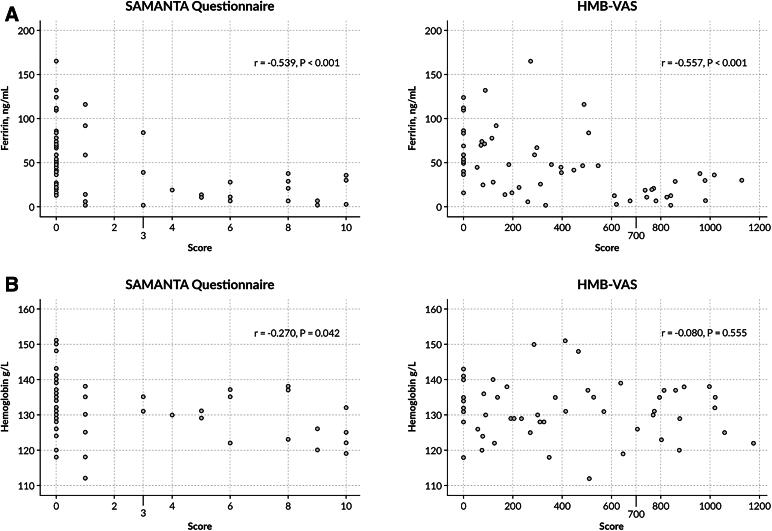
Correlation between the median serum ferritin **(A)** and hemoglobin **(B)** levels and the scores of the diagnostic tools SAMANTA questionnaire and HMB-VAS.

A significant negative correlation was observed between the serum ferritin levels and the score of both HMB diagnostic tools: *r* = −0.539 for the SAMANTA questionnaire and *r* = −0.557 for the HMB-VAS, both *p* < 0.001 (Fig. 2A). The serum Hb levels showed a negative correlation with the SAMANTA questionnaire scores (*r* = −0.270, *p* = 0.042), but not with the HMB-VAS tool scores (*r* = −0.080, *p* = 0.555) (Fig. 2B).

Given these results, the ferritin levels were used to define ID subsequently.

### Performance of the HMB diagnostic tools to identify ID

**FIG. 3.**
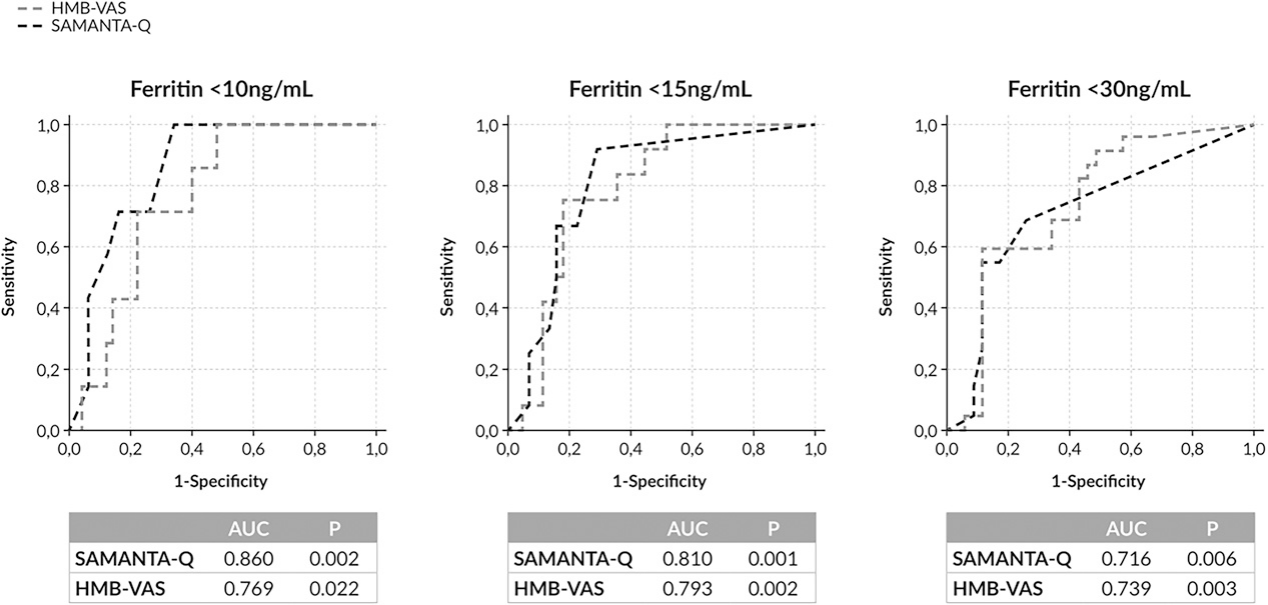
AUCs for the diagnostic tools SAMANTA questionnaire and HMB-VAS according to three ferritin thresholds. AUC, area under the curve.

The AUCs for the SAMANTA questionnaire and the HMB-VAS, according to the three ferritin thresholds used, are shown in Figure 3. The best sensitivity and specificity to identify ID in women with HMB using the SAMANTA questionnaire (score ≥3 points) was observed with the ferritin threshold of <10 ng/mL (sensitivity 71.4% [Confidence Interval (CI) 95% 35.2–93.5] and specificity 78% [CI 95% 65.2–87.7]) (Table 2). The best sensitivity and specificity to identify ID in these women (although low) using the HMB-VAS tool (score ≥700 points) was observed with the ferritin threshold of <15 ng/mL (sensitivity 58.3% [31.2–87.7] and specificity 82.2% [69.2–91.2]) (Table 2).

**Table 2.** Sensitivities and Specificities of the SAMANTA Questionnaire and the HMB-VAS Diagnostic Tools to Identify ID According to Three Ferritin Levels

HMB diagnostic tool	Ferritin level (ng/mL)	Sensitivity (%) [CI 95%]	Specificity (%)[CI 95%]
SAMANTA-Q (HMB when ≥3 points)	<10	71.4 [35.2–93.5]	78.0 [65.2–87.7]
	<15	66.7 [38.8–87.5]	82.2 [69.2–91.2]
	<30	54.5 [34.3–73.7]	88.6 [75.1–96.0]
HMB-VAS (HMB when ≥700 points)	<10	57.1 [23.5–86.1]	78.0 [65.2–87.7]
	<15	58.3 [31.2–87.7]	82.2 [69.2–91.2]
	<30	50.0 [30.2–69.8]	88.6 [75.1–96.0]

HMB, heavy menstrual bleeding; HMB-VAS, Heavy Menstrual Bleeding–Visual Analog Scale; ID, iron deficiency; SAMANTA-Q, SAMANTA questionnaire.

### QoL in women with and without HMB

**FIG. 4.**
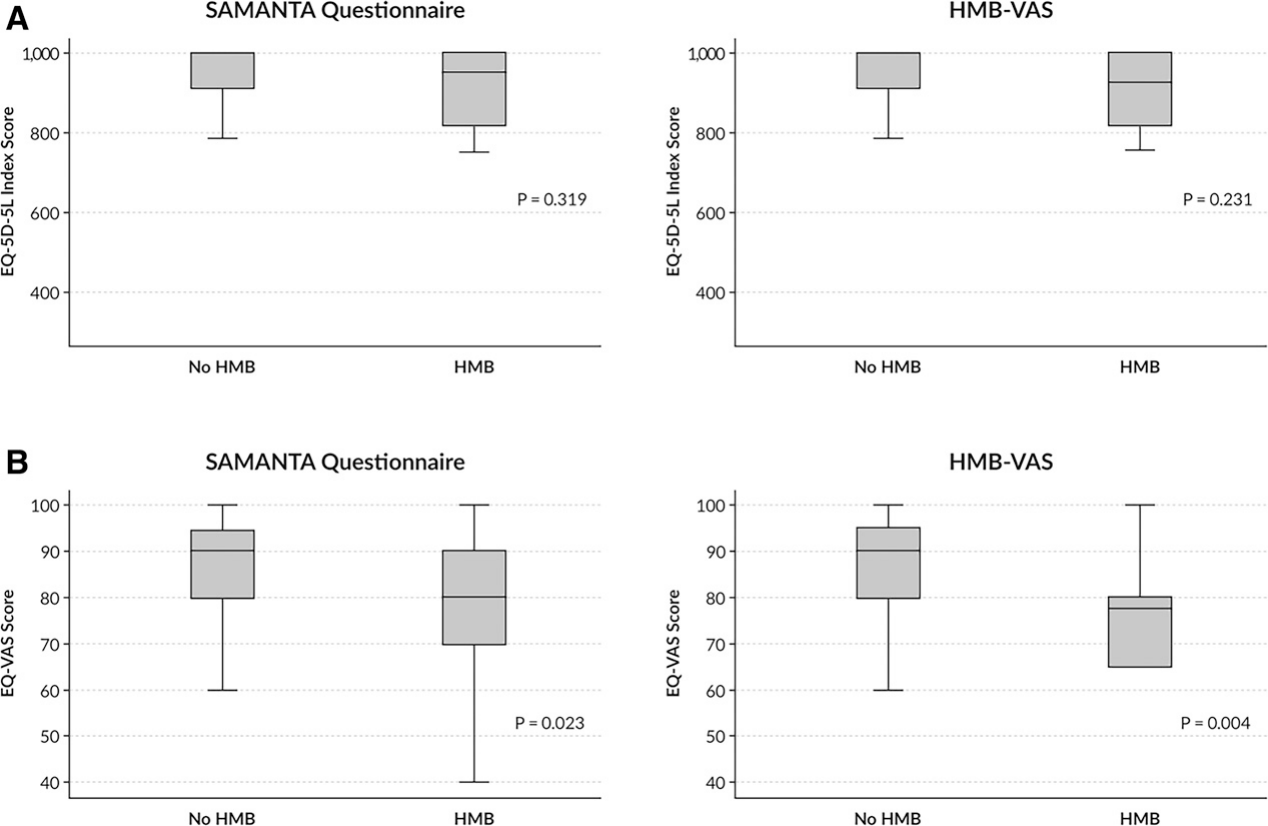
Median EQ-5D-5L index **(A)** and EQ-VAS **(B)** values in women with or without HMB according to the diagnostic tools SAMANTA questionnaire and HMB-VAS. EQ-5D-5L, EuroQoL five-dimensions five levels.

The median EQ-5D-5L index value was similar in women with and without HMB according to the SAMANTA questionnaire (0.96 [IQR 0.82–1] vs. 1 [0.91–1], *p* = 0.319) and to the HMB-VAS tool (0.93 [0.81–1.0] vs. 1.0 [0.91–1.0], *p* = 0.231), both respectively (Fig. 4A). The median EQ-VAS score was significantly lower in women with HMB versus those without HMB according to the SAMANTA questionnaire (80 [68.7–90] vs. 90 [80–95], *p* = 0.023) and the HMB-VAS tool (77.5 [65–81.2] vs. 90 [80–95], *p* = 0.004), both respectively (Fig. 4B).

No correlation was observed between the EQ-5D-5L index values and the scores of the SAMANTA questionnaire (*r* = −0.138, *p* = 0.306) or the HMB-VAS tool (*r* = −0.046, *p* = 0.733). A significant negative correlation was observed between the EQ-VAS score and the scores of both HMB diagnostic tools: *r* = −0.308, *p* = 0.02 for the SAMANTA questionnaire, and *r* = −0.294, *p* = 0.026 for the HMB-VAS tool.

## Discussion

The results of our study in IUD users with and without HMB show that the diagnosis of HMB using the SAMANTA questionnaire or the HMB-VAS tool is associated with lower serum ferritin levels (but not lower Hb levels), which correlate negatively with the scores of both HMB tools. However, only the SAMANTA questionnaire (HMB if ≥3 points) showed a relatively good performance for ID identification in women with HMB when ID was defined as a ferritin level of <10 ng/mL, thus identifying the more severe cases of ID. The scores of both HMB diagnostic tools correlated negatively with the EQ-VAS tool scores but not with those of the EQ-5D-5L index values.

ID/IDA in women with HMB is underdiagnosed and, therefore, undertreated in great part due to the lack of consensus among guidelines concerning the need to assess ID/IDA, how to do it (Hb vs. ferritin), and when.^9^ In a recent review of 22 clinical guidelines on ID and IDA management in women with HMB, Mansour et al.^9^ found that only half of these guidelines provided recommendations on iron determination, of which only three recommended a routine assessment of iron levels, advising the use of serum ferritin as an indicator of iron status.^23–25^ Noteworthy, four guidelines explicitly advised against this procedure based on the limitations of serum ferritin testing. Not only do these findings highlight the urgent need to promote serum ferritin assessment in women with HMB, but they also advocate for supporting the availability of easy-to-use screening tools. The results of our study, despite being exploratory, support using an easy-to-use HMB diagnostic tool such as the SAMANTA questionnaire—validated in Spanish^4^ and British English^5^—as a screening tool for ID in these women. To our knowledge, this is the first approach to identify a tool for screening patients in whom a ferritin measurement would be strongly recommended. Conversely to the SAMANTA questionnaire, the HMB-VAS showed limited performance in identifying ID in women with HMB, according to this diagnostic tool. The different ways to answer the questions posed (“yes–no” in the SAMANTA questionnaire for the six questions and a continuous scoring from 0 to 100 in both VAS scales of the HMB-VAS) may have contributed to the different performance of these tools to identify ID.

Another controversy in the diagnosis of ID/IDA is the lack of harmonization to define the ferritin threshold used for this diagnosis. Notably, Mansour et al.^9^ also found that of the 22 guidelines assessed, only four proposed serum ferritin thresholds for ID/IDA diagnosis, with <15 µg/L (as recommended by the World Health Organization for apparently healthy individuals older than five years old)^26^ and <30 µg/L being consistent with ID. This is likely due to the lack of good-quality studies to justify serum ferritin cutoffs to define absolute ID^27^ and the increase of ferritin levels in inflammatory conditions, even in those hidden.^26^ In one of these, Munro et al.^28^ recommend a threshold of <30 ng/mL for ID and <10 ng/mL for IDA. In our setting, 10 ng/mL is the lowest value within the serum ferritin value range considered normal in women (10–150 ng/mL). When assessing the performance of both HMB tools to screen for ID in women with HMB considering these three ferritin thresholds, only the SAMANTA questionnaire showed good performance, and this was for a ferritin level <10 ng/mL (sensitivity 71.4% and specificity 78.0%). The sensitivity of this HMB diagnostic tool decreased with increasing threshold at the expense of a greater specificity. This low discriminating ferritin value is likely due to the reduced sample size despite ferritin levels following a quasi-normal distribution in the population analyzed. However, the ROC curves suggest a potential good performance in the three ferritin levels analyzed.

We also proved the correlation between the SAMANTA questionnaire or the HMB-VAS scores and the EQ-VAS scores, thus providing evidence on how these HMB diagnostic tools capture generic QoL. Noteworthy, we only observed a relationship with this component of the EQ-5D-5L, but not with the index value. This finding is probably related to the ease of using the VAS scale to express degrees for the responses to the questions posed.^29^ Also, the tendency to provide socially desirable responses is lower with VAS scales than with categorical psychometric scales, translating into results closer to the respondents’ true attitudes.^29,30^

This post hoc analysis has some limitations. The fact that the study was not designed for the objective of our analysis and the reduced sample size (57 women) determines that our findings should be interpreted as exploratory and not yet conclusive. The value of the SAMANTA questionnaire to screen for HMB is currently being supported by different studies in heterogeneous populations.^31,32^ Its definite value for ID identification is to be validated in prospective studies with a larger sample size considering different thresholds as locally followed in clinical practice. Besides being validated in Spanish^4^ and British English,^5^ it is currently being validated in other languages and adolescent populations.

## Conclusions

This exploratory study shows the promising potential of the SAMANTA questionnaire for ID screening, providing an easy-to-use solution to an underdiagnosed clinical problem. The value of this HMB diagnostic tool and the HMB-VAS tool for ID identification should be further determined in well-designed studies. Both questionnaires significantly correlated with the EQ-VAS, thus demonstrating their value in assessing both components of HMB: the quantity of blood loss and its impact on QoL.

## Abbreviations Used

AUC: Area Under the Curve
BMI: Body Mass Index
CI: Confidence Interval
COLIBRI: Cooper and Levonorgestrel IUD Barcelona Research Initiative
Cu380-IUD: Nova T copper 380 mm² intrauterine device
EQ-5D-5L: EuroQoL Five-Dimension Five-Level
EQ-VAS: EuroQoL Visual Analog Scale
Hb: Hemoglobin
HMB: Heavy Menstrual Bleeding
HMB-VAS: Heavy Menstrual Bleeding–Visual Analog Scale
ID: Iron Deficiency
IDA: Iron Deficiency Anemia
IQR: Interquartile Range
IUD: Intrauterine Device
LNG13.5-IUD: Levonorgestrel 13.5 mg intrauterine device
QoL: Quality of Life
ROC: Receiver Operating Characteristic
SAMANTA: SAngrado Menstrual AbundaNTe en ginecologíA
SD: Standard Deviation
STROBE: Strengthening the Reporting of Observational Studies in Epidemiology
VAS: Visual Analog Scale
VASImp: Visual Analog Scale for Impact on activities of daily living
VASInt: Visual Analog Scale for Intensity of menstrual bleeding
